# Arkas: Rapid reproducible RNAseq analysis

**DOI:** 10.12688/f1000research.11355.2

**Published:** 2017-06-21

**Authors:** Anthony R. Colombo, Timothy J. Triche Jr, Giridharan Ramsingh

**Affiliations:** 1 Jane Anne Nohl Division of Division of Hematology and Center for the Study of Blood Diseases, Keck School of Medicine of University of Southern California, Los Angeles, CA, 90033, USA

**Keywords:** transcriptome, sequencing, RNAseq, automation, cloud computing

## Abstract

The recently introduced Kallisto pseudoaligner has radically simplified the quantification of transcripts in RNA-sequencing experiments.  We offer cloud-scale RNAseq pipelines *Arkas-Quantification*, and *Arkas-Analysis *available within Illumina’s BaseSpace cloud application platform which expedites Kallisto preparatory routines, reliably calculates differential expression, and performs gene-set enrichment of REACTOME pathways *.  *Due to inherit inefficiencies of scale, Illumina's BaseSpace computing platform offers a massively parallel distributive environment improving data management services and data importing. *Arkas-Quantification *deploys Kallisto for parallel cloud computations and is conveniently integrated downstream from the BaseSpace Sequence Read Archive (SRA) import/conversion application titled *SRA Import*. *Arkas-Analysis *annotates the Kallisto results by extracting structured information directly from source FASTA files with per-contig metadata, calculates the differential expression and gene-set enrichment analysis on both coding genes and transcripts. The *Arkas* cloud pipeline supports ENSEMBL transcriptomes and can be used downstream from the *SRA Import* facilitating raw sequencing importing, SRA FASTQ conversion, RNA quantification and analysis steps.

## Introduction

High-performance computing based bioinformatic workflows have three main subfamilies: in-house computational packages, virtual-machines (VMs), and cloud based computational environments. The in-house approaches are substantially less expensive when raw hardware is in constant use and dedicated support is available, but internal dependencies can limit reproducibility of computational experiments. Specifically, “superuser’” access needed to deploy container-based, succinct code encapsulations (often referred to as "microservices" elsewhere) can run afoul of normal permissions, and the maintenance of broadly usable sets of libraries across nodes for users can lead to shared code dynamically linking to different libraries under various user environments. By contrast, modern cloud-based approaches and parallel computing are forced by necessity to offer a user-friendly platform with high availability to the broadest audience. Platform-as-a-service approaches take this one step further, offering controlled deployment and fault tolerance across potentially unreliable instances provided by third parties such as Amazon Web Service Elastic Compute Cloud (AWS EC2) and enforcing a standard for encapsulation of developers' services such as Docker. Within this framework, the user or developer cedes some control of the platform and interface, in exchange for the platform provider handling the details of workflow distribution and execution. This has provided the best compromise of usability and reproducibility when dealing with general audiences. In this regard, the lightweight-container approach exemplified by Docker lead to rapid development and deployment compared to VMs. Combined with versioning of deployments, it is feasible for users to reconstruct results from an earlier point in time, while simultaneously re-evaluating the generated data under state-of-the-art implementations.

Docker offers advantages for reproducible research practices, and also is the principal infrastructure to leading platforms such as Illumina's BaseSpace platform, Google Genomics, Galaxy and SevenBridges. Cloud computational ecosystems preserve developmental environments using Docker containerization framework, and improves bioinformatic validation. Containerized cloud applications form part of the global distributive effort and are favorable over local in-house computational pipelines because they offer rapid access to numerous public workflows, easy interfacing to archived read databases, and accelerate the upholding process of raw data.

A major bottleneck in RNAseq analysis is the processing steps for importing raw data. The majority of RNAseq analysis pipelines consist of read preparation steps, followed by computationally expensive alignment against a reference. Software for calculating transcript abundance and assembly can surpass 30 hours of computational time ^1^. If known or putative transcripts of defined sequences are the primary interest, then pseudoalignment, which is defined as near-optimal RNAseq transcript quantification, is achievable in minutes on a standard laptop using Kallisto software ^1^. *Arkas* was developed using a simple framework, yet massively parallel, for RNAseq transcript quantification that would allow users to expedite pseudoalignment on arbitrary datasets, and significantly reduce the amount of required preparatory routines.

 In collaboration with Illumina (San Diego, USA) the available BaseSpace platform was already well-suited for parallel transformation of raw sequencing data into analytical results. BaseSpace has an available application *SRA Import* which automates SRA importing and FASTQ conversion pre-processing steps. The application *SRA Import* is simple requiring the SRA accession number and limits imports to 25gb per application call. *Arkas* can ingest successfully imported samples avoiding all raw data handling. For example, if one were interested in re-analyzing an experiment from SRA with reads totaling 141.3GB, *Arkas* facilitates SRA processing and state-of-the-art pseudoalignment by reducing raw sequencing data to summary quantifications totaling 1.63GB and includes an extensive analysis report of less than 10MB. The total data reduction exceeds 4 orders of magnitude with little or no loss of user-visible information. Moreover, the untouched original data is never discarded unless the user explicitly demands it. The appropriate placement of *Arkas* applications adjacent to the origin of sequencing data removes cumbersome data relocation costs and greatly facilitates sequencing archive re-analysis using state-of-the-art pseudoalignment.

*Arkas*, encapsulates Kallisto, automates the construction of composite transcriptomes from, quantifies transcript abundances, and implements reproducible rapid differential expression analysis coupled with gene set enrichment analysis. The *Arkas* workflow is versionized into Docker containers and publicly deployed within Illumina's BaseSpace platform which ingests raw RNA sequencing data and completes a full analysis in approximately 2 hours.

## Methods

The first step in the *Arkas* pipeline requires *Arkas-Quantification* to transform all the raw RNA sequencing data of an entire experiment into Kallisto pseudoaligned quantification output data. The second step, *Arkas-Analysis*, requires the pseudoaligned data to be input with respect to a comparison and control group, and returns a comprehensive analysis including differential expression, and gene-set enrichment.

If the user selects the defaults, *Arkas-Quantification* will complete pseudoalignment in approximately 43–60 minutes. *Arkas-Quantification* completion time is independent of the number of samples input, but is restricted to node availability (AWS EC2 node availability is fairly high). *Arkas-Analysis,* will complete using a single node in approximately 1–1.5 hours for moderate sample group sizes (N ≤ 20), and under 2–2.5 hours for much larger designs.

### Arkas-Quantification implementation

Arkas is a two-step cloud pipeline. *Arkas-Quantification* is the first step, which reduces the computational steps required to quantify and annotate large numbers of samples against large catalogs of transcriptomes. *Arkas-Quantification* calls Kallisto for on-the-fly transcriptome indexing and quantification recursively for numerous sample directories. Kallisto quantifies transcript abundance from input RNAseq reads by using pseudoalignment, which identifies the read-transcript compatibility matrix ^1 ^. The compatibility matrix is formed by counting the number of reads with the matching alignment; the equivalence class matrix has a much smaller dimension compared to matrices formed by transcripts and read coverage. Computational speed is gained by performing the Expectation Maximization (EM) algorithm over a smaller matrix.

For RNAseq projects with many sequenced samples, *Arkas-Quantification* encapsulates expensive transcript quantification preparatory routines, while uniformly preparing Kallisto execution commands within a versionized environment encouraging reproducible protocols. The quantification step automates the index caching, annotation, and quantification associated while running the Kallisto pseudoaligner integrated within the BaseSpace environment. For users interested in quality control checks, BaseSpace offers an independent application *FastQC* which performs fastqc on sequencing data. The first step in the pipeline can process raw reads into transcript and pathway collection results within Illumina’s BaseSpace cloud platform, quantifying against default transcriptomes such as ERCC spike-ins, ENSEMBL non-coding RNA, or cDNA build 88 for both *Homo sapiens* and *Mus musculus*; further the first step supports user uploaded FASTA files for customized analyses. *Arkas-Quantification* can support microRNAs (miRNA), however we encourage users to analyze miRNAs separately because pseudoalignment requires reducing k-mer size in the Target-DeBruijn Graph (TDBG) to miRNA sequence lengths (ranging from 16–22) which can increase path ambiguities. *Arkas-Quantification* is packaged into a Docker container and is publicly available as a cloud application within BaseSpace.

### Arkas-Analysis implementation

Previous work ^2^ has revealed that filtering transcriptomes to exclude lowly-expressed isoforms can improve statistical power, while more-complete transcriptome assemblies improve sensitivity in detecting differential transcript usage. Based on earlier work by Bourgon *et al.* ^3^, we included this type of filtering for both gene- and transcript-level analyses within *Arkas-Analysis*. The analysis pipeline automates annotations of quantification results, resulting in more accurate interpretation of coding and transcript sequences in both basic and clinical studies by just-in-time annotation and visualization.

**Figure 1.**
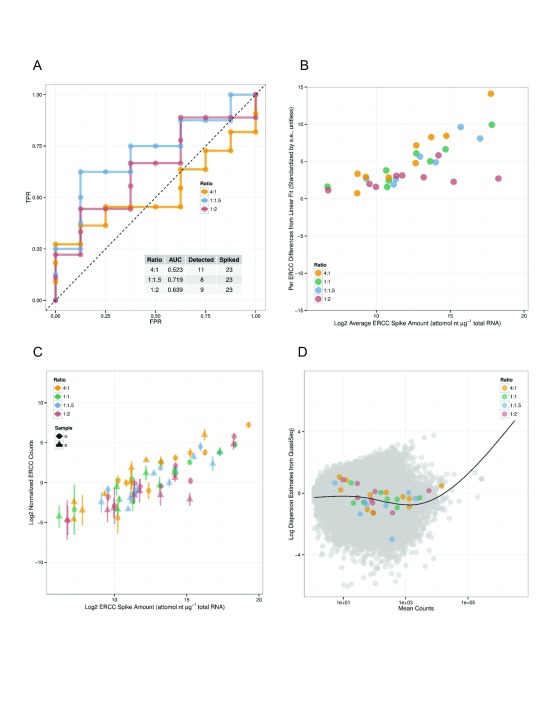
*Arkas-Analysis* ERCC spike-in Controls Report. **A**) The Receiver Operator Characteristic plot of (detected) ERCC ratios in gene expression experiments. The X-axis shows the False Positive Rate, the Y-axis shows True Positive Rate. **B**) and **C**) shows the dynamic range of abundances of ERCC RNA amounts with a linear model fit, and ERCC RNA counts. **D**) shows a dispersion of mean transcript abundances and the estimated dispersion.

*Arkas-Analysis* integrates quality control analysis for experiments that include Ambion spike-in controls, multiple normalization selections for both coding gene and transcript differential expression analysis, and differential gene-set analysis. If ERCC spike-ins, defined by the External RNA Control Consortium ^4^, are detected then *Arkas-Analysis* will calculate Receiver Operator Characteristic (ROC) plots using 'erccdashboard' ^5^. The ERCC analysis reports average ERCC Spike amount volume, comparison plots of ERCC volume amount, and normalized ERCC counts ( Figure 1).

Subsequent analyses import the data structure from SummarizedExperiment and creates a sub-class titled KallistoExperiment that preserves the S4 structure and is convenient for handling assays, phenotypic and genomic data. KallistoExperiment includes GenomicRanges ^6^, preserving the ability to handle genomic annotations and alignments, supporting efficient methods for analyzing high-throughput sequencing data. The KallistoExperiment sub-class serves as a general-purpose container for storing feature genomic intervals and pseudoalignment quantification results against a reference genome called by Kallisto. By default KallistoExperiment couples assay data such as the estimated counts, effective length, estimated median absolute deviation, and transcript per million count where each assay data is generated by a Kallisto run; the stored feature data is a GenomicRanges object from 6, storing transcript length, GC content, and genomic intervals.

**Figure 2.**
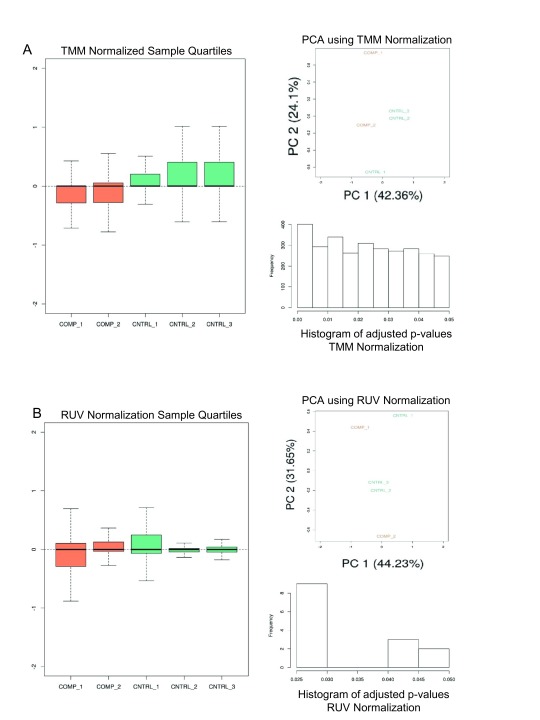
*Arkas-Analysis* Normalization Report: Normalization Analysis Using TMM and RUV. **A**) TMM normalization is performed on sample data and depicts the sample quantiles on normalized sample expression, PCA plot, and histogram of the adjusted p-values calculated from the DE analysis. Orange is the comparison group and green is the control group. **B**) A similar analysis is performed with RUV in-silico normalization.

**Figure 3.**
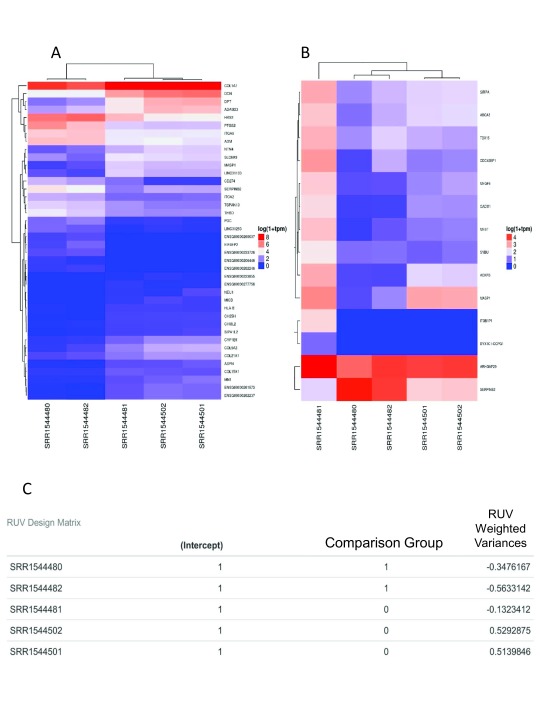
*Arkas-Analysis* Differential Expression Report: DE using TMM and RUV. **A**) DE analysis using TMM normalization. The X-axis is the sample names (test data), the Y-axis are Gene symbols (HUGO). Expression values are plotted in log _10_ 1+TPM. **B**) Similar analysis using RUV normalization. **C**) The design matrix with the RUV adjusted weights. The sample names are test data used in demonstrating the general analysis report output.

Given a KallistoExperiment containing the Kallisto sample abundances, principal component analysis (PCA) is performed ^7^ on trimmed mean of M-value (TMM) normalized counts ^8^ ( Figure 2A). Differential expression (DE) is calculated on the library normalized transcript expression values, and the aggregated transcript bundles of corresponding coding genes using limma/voom linear model ^9^ ( Figure 3A). In addition to library normalization, we wished to add an optional data driven normalization. In the analysis pipeline, an unsupervised normalization method would not require more than a two group experimental design which was favorable due to its simplicity. Alternatively, supervised data driven normalization is a specialized task which requires users to define batch groups, and/or additional experimental groups. Further, the adjusted data must be evaluated in the context of the experiment. In-silico normalization, using factor analysis, effectively removes unwanted variation driven entirely by data ^10^.

The analysis report returns comprehensive visualization results. PCA and DE analysis of both transcripts and coding genes is performed with easily interpretable images ( Figure 2B, Figure 3B, Figure 3C). In each DE analysis FDR filtering method is defaulted to 'Benjamini-Hochberg', if there are no resultant DE genes/transcripts the FDR methods is switched to 'none'. *Arkas-Analysis* consumes the Kallisto data output from *Arkas-Quantification*, and automates DE analysis using TMM normalization and in-silico normalization on both transcript and coding gene expression in a defaulted two group experimental design, allowing customized selections. One must examine and compare the PCA sample clustering, and sample boxplots between the two methods to determine the improvement of in-silico normalization ( Figure 2). If RUV improves the PCA clustering within the context of an experiment, and reduces the number of outliers observed in boxplots then it is likely that the normalization weights are useful.

**Figure 4.**
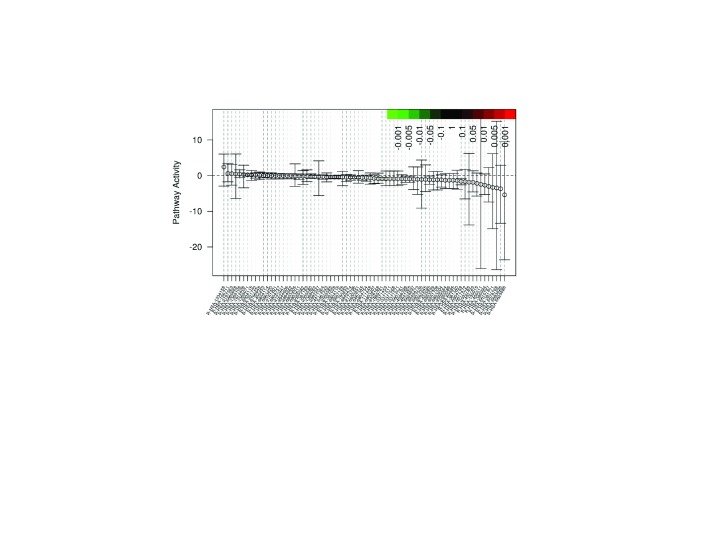
*Arkas-Analysis* Gene-Set Enrichment Plot. Gene-Set enrichment output report, each point represents the differential mean activity of each gene-set with 95% confidence intervals. The X-axis are individual gene-sets. The Y-axis is the log _2_ fold change.

Gene set differential expression, which includes gene-gene correlation inflation corrections, is calculated using Qusage ^11^. Qusage calculates the variance inflation factor, which corrects the inter-gene correlation that results in high type 1 errors using pooled or non-pooled variances between experimental groups. The gene set enrichment is conducted using Reactome pathways constructed using ENSEMBL transcript/gene identifiers ( Figure 4 and Table 1); REACTOME gene sets are not as large as other databases, so *Arkas-Analysis* outputs DE analysis in formats compatible with more exhaustive databases such as Advaita. The DE files are compatible as a custom upload into Advaita iPathway guide, which offers an extensive Gene Ontology (GO) pathway analysis. Pathway enrichment analysis can be performed from the BaseSpace cloud system downstream from parallel differential expression analysis and can integrate with other pathway analysis software tools.

**Table 1.** *Arkas-Analysis* Gene-Set Enrichment Statistics. The columns represent the Reactome pathway name corresponding to the depicted pathways in Figure 4, the log _2_fold change, p-value, adjusted FDR, and an active link to the Reactome website with visual depictions of the gene/transcript pathway. *Arkas-Analysis* will output a similar report testing transcript-level sets.

Pathway name	Log fold change	P.value	FDR	Gene URL
R-HAS-1989781	-0.87	0.0008	0.06	http://www.reactome.org/PathwayBrowser/#/R-HSA-1989781
R-HAS-2173796	-0.51	0.007	0.217	http://www.reactome.org/PathwayBrowser/#/R-HSA-2173796
R-HAS-6804759	-1.62	0.009	0.217	http://www.reactome.org/PathwayBrowser/#/R-HSA-6804759
R-HAS-381038	-0.43	0.013	0.226	http://www.reactome.org/PathwayBrowser/#/R-HSA-381038
R-HAS-2559585	-0.4	0.032	0.341	http://www.reactome.org/PathwayBrowser/#/R-HSA-2559585
R-HAS-4086398	-0.95	0.033	0.341	http://www.reactome.org/PathwayBrowser/#/R-HSA-4086398
R-HAS-4641265	-0.95	0.033	0.341	http://www.reactome.org/PathwayBrowser/#/R-HSA-4641265
R-HAS-422085	-1.17	0.04	0.361	http://www.reactome.org/PathwayBrowser/#/R-HSA-422085
R-HAS-5467345	-0.56	0.069	0.389	http://www.reactome.org/PathwayBrowser/#/R-HSA-5467345
R-HAS-6804754	-0.57	0.07	0.389	http://www.reactome.org/PathwayBrowser/#/R-HSA-6804754
R-HAS-6803204	-1.19	0.081	0.389	http://www.reactome.org/PathwayBrowser/#/R-HSA-6803204

### Data variance between software versions

**Figure 5.**
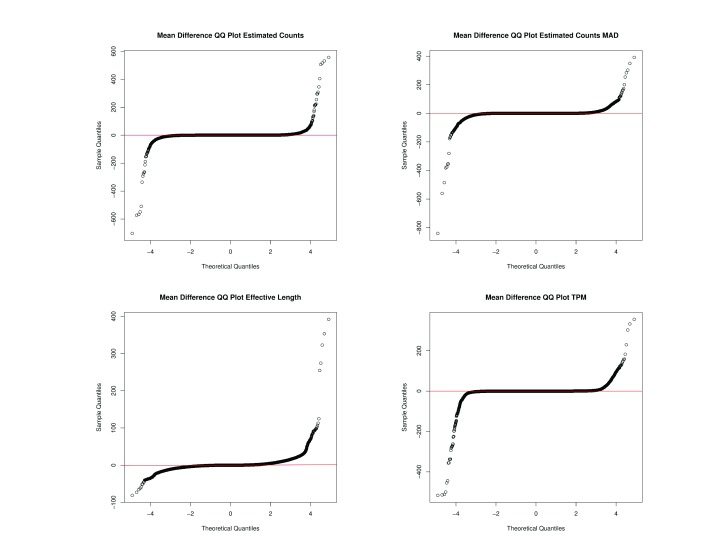
Quantile-Quantile Plots of Data Variation Comparing Differences in Kallisto Data from Versions 0.43.1 and 0.43.0. The X-axis depicts the theoretical quantiles of the standardized mean differences. The Y-axis represents the observed quantiles of standardized mean differences.

We wished to show the importance of enforcing matching versions of Kallisto when quantifying transcripts because there is deviation of data between versions. Due to updated versions and improvements of Kallisto software, there obviously exists variation of data between algorithm versions ( Figure 5, Supplementary Table 1, Supplementary Table 2). We calculated the standardized mean differences, and the variation of the differences between the same 5 samples from Kallisto (setting bootstraps = 42) versions 0.43 and 0.43.1 ( Supplementary Table 2), and found large variation of differences between raw values generated by differing Kallisto versions, signifying the importance of version analysis of Kallisto results.

Supplementary Table 1 shows the variation of the errors of the raw values such as estimated counts, effective length, and estimated median absolute deviation using the same Kallisto version 0.43.0. As expected, Kallisto data generated by the same Kallisto version had very low variation of errors within the same version 0.43.0 for every transcript across all samples. However, upon comparing Kallisto version 0.43.1 to version 43.0 using the raw data such as estimate abundance counts, effective length, estimated median absolute deviation, and transcript per million values, we found, as expected, large variation of data.

Supplementary Table 2 shows that there is large variation of the differences of Kallisto data calculated between differing versions. Figure 5 depicts the standardized mean differences, i.e. errors, between Kallisto versions fitted to a theoretical normal distribution. The quantile-quantile plots show that the errors are marginally normal, with a consistent line centered near 0 but also large outliers ( Figure 5). As expected, containerizing analysis pipelines will enforce versionized software, which benefits reproducible analyses.

The Dockerization of Arkas BaseSpace applications versionizes the Kallisto reference index to enforce that the Kallisto software versions are identical, and further documents the Kallisto version used in every cloud analysis. The enforcement of reference versions and Kallisto software versions prevents errors when comparing experiments.

### Operation

*Arkas-Quantification* instructions are provided within BaseSpace (details for new users can be found here). *Arkas* is a web style format, but can also be launched using the command line using BaseSpace Command Line Interface. The inputs are RNA sequencing samples, which may include SRA imported reads, and the outputs include the Kallisto data, .tar.gz files of the Kallisto sample data, and a report summary ( Supplementary Figure 1 and Supplementary Figure 2). Users may select for species type ( *Homo sapiens* or *Mus musculus*), optionally correct for read length bias, and optionally select for the generation of pseudoBAMs. More significantly, users have the option to use the default transcriptome (ENSEMBL build 88) or to upload a custom FASTA of their choosing. For users that wish for local analysis, they can download the sample .tar.gz Kallisto files and analyze the data locally.

The *Arkas-Analysis* instructions are provided within the BaseSpace environment. The input for the analysis app is the *Arkas-Quantification* sample data, and the output files are separated into corresponding folders. The analysis also depicts figures for each respective analysis ( Figure 1– Figure 4) and the images can be downloaded as a HTML format.

### Annotation of coding genes and transcripts

The extraction of genomic and functional annotations directly from FASTA contig comments, eliding sometimes-unreliable dependencies on services such as BioMart, are calculated rapidly. The annotations were performed with a run time of 2.336 seconds ( Supplementary Table 3) which merged the previous Kallisto data from 5 samples, creating a KallistoExperiment class with feature data containing a GenomicRanges ^11^ object with 213782 ranges and 9 metadata columns. The system runtime for creating a merged KallistoExperiment class for 5 samples was 23.551 seconds ( Supplementary Table 4).

## Discussion

### Complete transcriptomes enrich annotation information, improving downstream analyses

The choice of catalog, and the type of quantification performed, influence the results of sequencing analysis. ENSEMBL reference genomes are provided to GENCODE as a merged database from Havana's manually curated annotations with ENSEMBL's automatic curated coordinates. AceView, UCSC, RefSeq, and GENCODE have approximately twenty thousand protein coding genes, however AceView and GENCODE have a greater number of protein coding transcripts in their databases. RefSeq and UCSC references have less than 60,000 protein coding transcripts, whereas GENCODE has 140,066 protein coding loci. AceView has 160,000 protein coding transcripts, but this database is not manually curated. GENCODE is annotated with special attention given to long non-coding RNAs (lncRNAs) and pseudogenes, improving annotations and coupling automated labeling with manual curating. The database selected for protein coding transcripts can influence the amount of annotation information returned when querying gene/transcript level databases.

Although previously overlooked, lncRNAs have been shown to share features and alternate splice variants with mRNA, revealing that lncRNAs play a central role in metastasis, cell growth and cell invasion ^12^. LncRNA transcripts have been shown to be functional and are associated with cancer prognosis. *Arkas’* default transcriptomes include ENSEMBL (build 88) cDNA and non-coding RNA reference sequences.

### Arkas cloud pipeline: modern and simple

Recent developments for virtualized operating systems, such as Docker, allow for local software environments to be preserved, whereas cloud platforms deploy the preserved software. Docker allows users to build layers of read/write access files, creating a portable operating system which exhaustively controls software versions and data, while systematically preserving the pipeline software. Currently, Docker is the principal infrastructure for cloud bioinformatic computational software platforms such as Illumina's BaseSpace platform, Google Genomics, SevenBridges, and Galaxy. 

The Google Cloud Platform supports popular languages such as Python, Node, and Ruby with services related to computing, and storage. Google Genomics Platform has a steeper learning curve recommending familiarity with services such as Compute Engine, and Cloud Storage. Google Genomics hosts cloud storage transfer services for importing source data to storage buckets from HTTP/HTTPS locations. Data management services outside the Google Genomics platform, such as Globus, serves SRA database which can interact with Google Genomics applications reducing the bottleneck of SRA downloads.

Google offers very cost effective means for analyzing data, but requires expensive preparatory routines. Tatlow *et al.* employed Kallisto to pseudoalign 12,307 RNA-sequencing samples by renting preemptible VMs from Google Cloud Platform for as little as $0.09 per sample. Tatlow *et al.* pseudoaligned 1,811 breast carcinoma samples completing on average in 101 minutes, and 934 Cancer Cell Line Encyclopedia (CCLE) BAM files completing in 84.7 minutes on average. However large scale efforts require specialized knowledge for controlling containers (e.g. Kubernetes), manage resources, and queues. Although Tatlow *et al.* skillfully employed a cost-effective implementation of RNA-Seq analysis of massive databases, they mention a critical need for reducing the preprocessing routines involved in cloud computing ^13^.

Galaxy offers shared workflows and analytical pipelines but is limited in the services related to storage due to the usage of public servers. In this light private storage platforms can flexibly store experimental data, although the range of analysis tools is not as wide compared to open-source platforms. Galaxy offers many usable tools with a wide range of visualization pipelines. In contrast, BaseSpace offers tools to accomplish specific tasks at the expense of lowering the learning curve, which may be attractive for researchers interested in immediate, and verifiable, results.

BaseSpace offers other RNAseq tools and another analysis pipeline *RNAExpress* which reduces preparatory routines *. RNAExpress* runs DESeq2 and can be used to cross validate *Arkas-Analysis*. DESeq2 uses a negative binomial distribution to model differential expression, whereas *Arkas* implements limma/voom empirical Bayes analysis pipeline. *RNAExpress* completed in 109 minutes comparing 4 controls and 4 comparison samples. Using the same samples, *Arkas-Quantification* completed in 42 minutes, and *Arkas-Analysis* completed in 54 minutes. Illumina’s BaseSpace catalog of modern, yet simple, tools are attractive for users wishing share sessions, and to rapidly (re)analyze entire experiment(s).

## Conclusion

*Arkas* integrates the Kallisto pseudoalignment algorithm into the BaseSpace cloud computation ecosystem that can implement large-scale parallel ultra-fast transcript abundance quantification. We reduce a computational bottleneck by freeing inefficiencies from utilizing rapid transcript abundance calculations and connecting accelerated quantification software to the Sequencing Read Archive. We remove the second bottleneck because we reduce the necessity of database downloading; instead we encourage users to download aggregated analysis results. We also expand the range of common sequencing protocols to include an improved gene-set enrichment algorithm, Qusage, and allow for exporting into an exhaustive pathway analysis platform, Advaita, over the AWS EC2 field in parallel.

## Data availability

The data referenced by this article are under copyright with the following copyright statement: Copyright: © 2017 Colombo AR et al.

### Data used in testing variation between versions

**Controls:** SRR1544480 Immortal-1

SRR1544481 Immortal-2

SRR1544482 Immortal-3

**Comparison:** SRR1544501 Qui-1

SRR1544502 Qui-2

## Software availability

Latest source code:

https://github.com/RamsinghLab/Arkas-RNASeq

Archived source code as at the time of publication:

DOI: 10.5281/zenodo.545654 ^21^

License:

MIT license

### Reference FASTA annotation files

For Homo-sapiens and Mus-musculus ENSEMBL FASTA files were downloaded here for release 88.

### ERCC sequences

The ERCC sequences are provided in a SQL database format located here

## Supplementary material

**Supplementary Table 1: Data variation with matching Kallisto versions.** This shows the variation of mean differences between data using the matching Kallisto version 0.43.0. The rows represent the samples from the first run using version 0.43.0. The columns represent the samples from an additional run with version 0.43.0.

.

**Supplementary Table 2: Data variation with non-matching Kallisto versions.** Variation of mean differences between non-matching Kallisto versions and a randomly selected run previously generated ( Supplementary Table 1). The rows are samples run using version 0.43.0, the columns are runs using version 0.43.1.

.

**Supplementary Table 3: Annotation runtime.** System runtime for full annotation of a merged KallistoExperiment (seconds). The columns represent system runtime, the Elapsed Time is the total runtime.

.

**Supplementary Table 4: KallistoExperiment formation runtime.** System runtime for the creation of a merged KallistoExperiment (seconds). The columns are similar to Supplementary Table 3.

.

**Supplementary Figure S1: *Arkas-Quantification* Web-Style user interface.** The input field for **A**) *Arkas-Quantification* and **B**) *Arkas-Analysis* demonstrating SRA re-quantification. The control and comparison samples were obtained using BaseSpace *SRA Import* application, and were input into the *Arkas* pipeline.

.

**Supplementary Figure S2: *Arkas-Quantification* output directory. A**) Depicts the *Arkas-Quantification* output directory which includes sample folders containing Kallisto data. **B**) Depicts the contents of a specific folder output by *Arkas-Quantification*.
