# Supplementary material for: Arkas: Rapid reproducible RNAseq analysis
Source: F1000Res. 2017 Jun 21;6:586. Originally published 2017 Apr 27. [Version 2] doi: 10.12688/f1000research.11355.2 (PMC5553089; doi:10.12688/f1000research.11355.2)
Supplement: Supplementary file 5 [file f1000research-6-12854-s0004.tgz › 30b449ba-29a9-46c3-8b32-9e39452552d5.pdf]

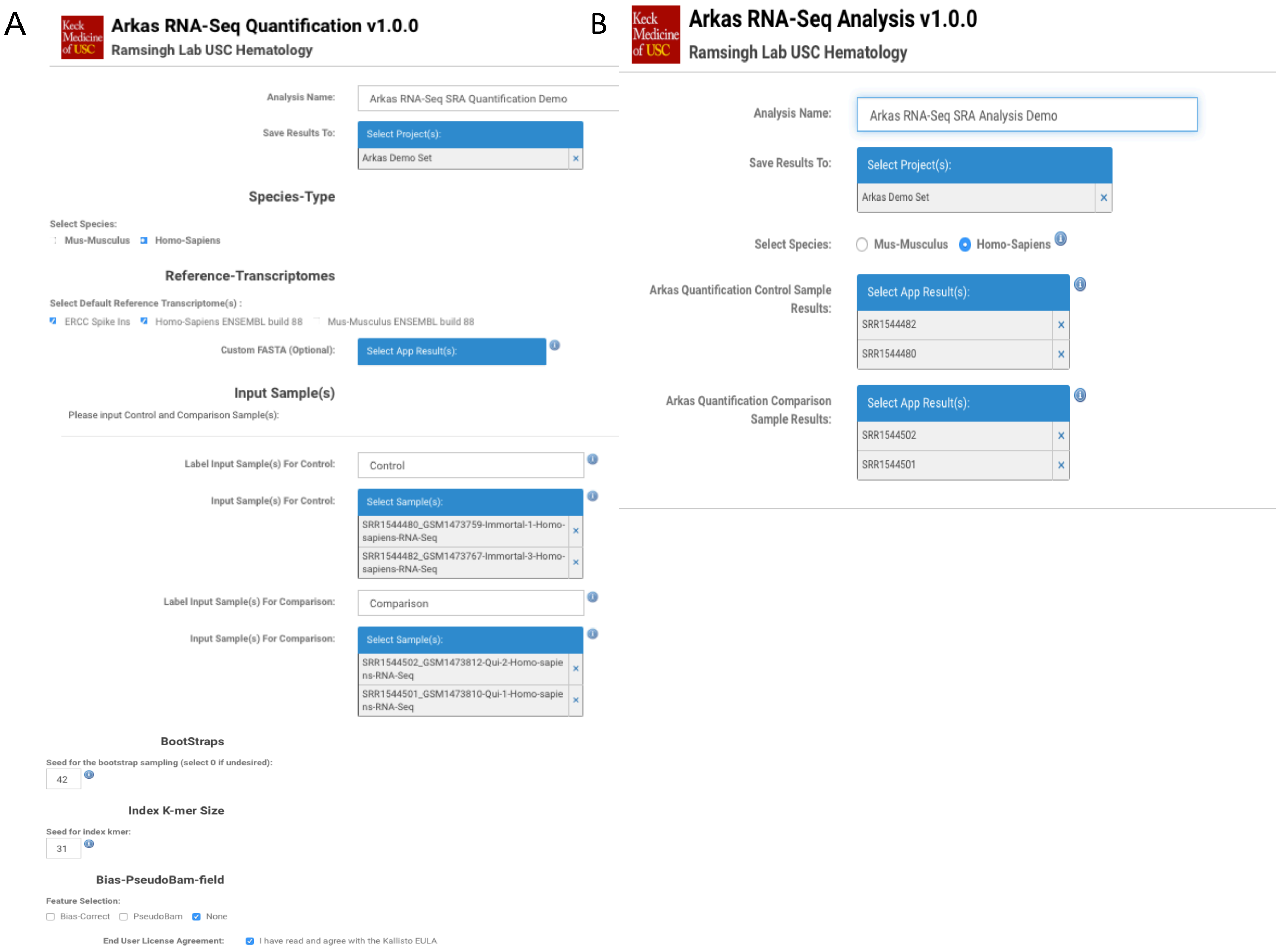
A
Arkas RNA-Seq Quantification v1.0.0
Ramsingh Lab USC Hematology
Analysis Name:
Arkas RNA-Seq SRA Quantification Demo
Save Results To:
Select Project(s):
Arkas Demo Set
Species-Type
Select Species:
Mus-Musculus
Homo-Sapiens
Reference-Transcriptomes
Select Default Reference Transcriptome(s) :
ERCC Spike Ins
Homo-Sapiens ENSEMBL build 88
Mus-Musculus ENSEMBL build 88
Custom FASTA (Optional):
Select App Result(s):
Input Sample(s)
Please input Control and Comparison Sample(s):
Label Input Sample(s) For Control:
Control
Input Sample(s) For Control:
Select Sample(s):
SRR1544480_GSM1473759-Immortal-1-Homo-sapiens-RNA-Seq
SRR1544482_GSM1473767-Immortal-3-Homo-sapiens-RNA-Seq
Label Input Sample(s) For Comparison:
Comparison
Input Sample(s) For Comparison:
Select Sample(s):
SRR1544502_GSM1473812-Qui-2-Homo-sapiens-RNA-Seq
SRR1544501_GSM1473810-Qui-1-Homo-sapiens-RNA-Seq
BootStraps
Seed for the bootstrap sampling (select 0 if undesired):
42
Index K-mer Size
Seed for index kmer:
31
Bias-PseudoBam-field
Feature Selection:
Bias-Correct
PseudoBam
None
End User License Agreement:
I have read and agree with the Kallisto EULA
B
Arkas RNA-Seq Analysis v1.0.0
Ramsingh Lab USC Hematology
Analysis Name:
Arkas RNA-Seq SRA Analysis Demo
Save Results To:
Select Project(s):
Arkas Demo Set
Select Species:
Mus-Musculus
Homo-Sapiens
Arkas Quantification Control Sample Results:
Select App Result(s):
SRR1544482
SRR1544480
Arkas Quantification Comparison Sample Results:
Select App Result(s):
SRR1544502
SRR1544501
