# Supplementary material for: Arkas: Rapid reproducible RNAseq analysis
Source: F1000Res. 2017 Jun 21;6:586. Originally published 2017 Apr 27. [Version 2] doi: 10.12688/f1000research.11355.2 (PMC5553089; doi:10.12688/f1000research.11355.2)
Supplement: Supplementary file 6 [file f1000research-6-12854-s0005.tgz › d88cfb22-3bd3-4781-a7d7-c24ce6f6250b.pdf]

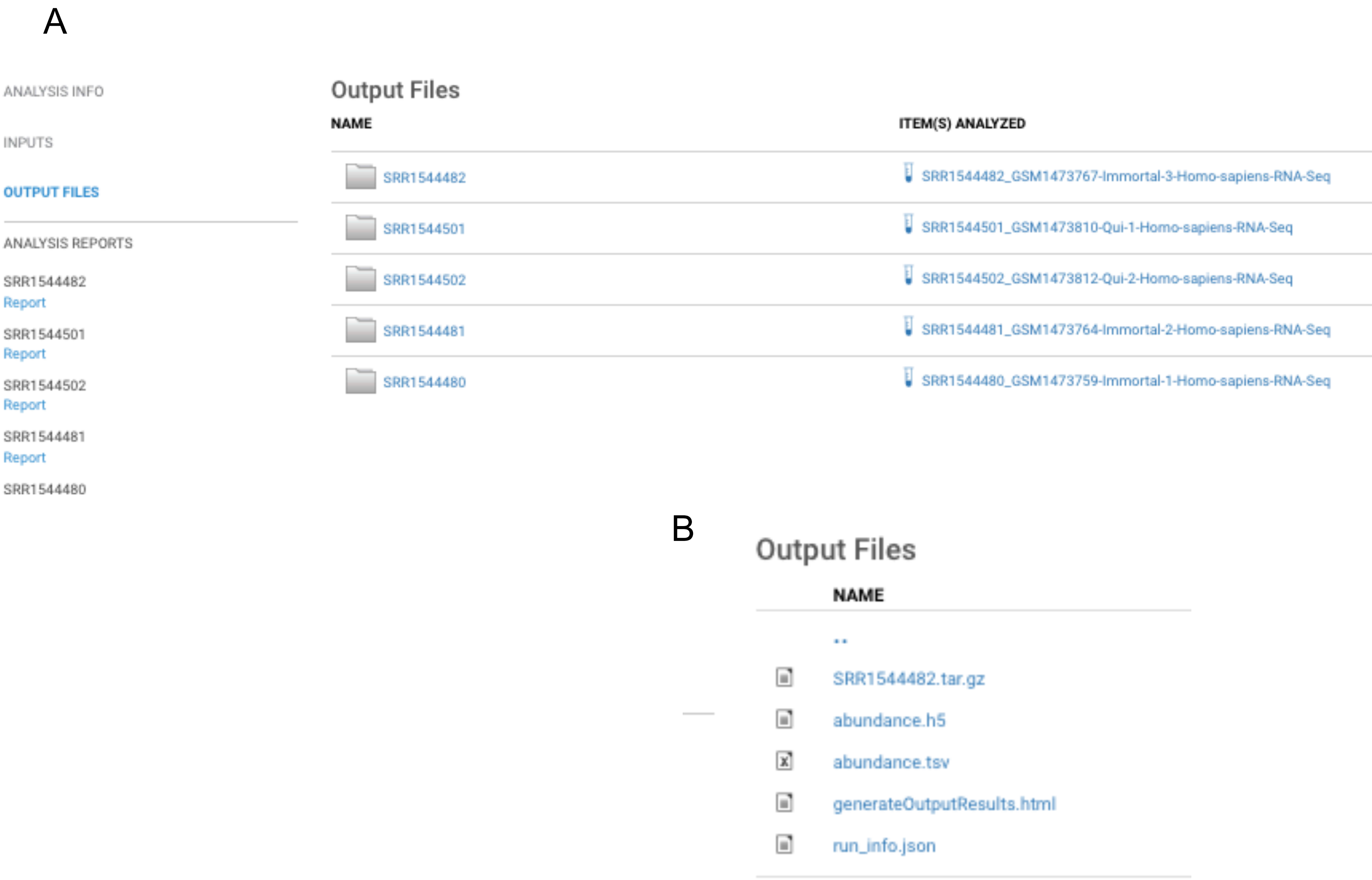
A
ANALYSIS INFO
INPUTS
OUTPUT FILES
ANALYSIS REPORTS
SRR1544482
Report
SRR1544501
Report
SRR1544502
Report
SRR1544481
Report
SRR1544480
Output Files
NAME
ITEM(S) ANALYZED
SRR1544482
SRR1544482_GSM1473767-Immortal-3-Homo-sapiens-RNA-Seq
SRR1544501
SRR1544501_GSM1473810-Qui-1-Homo-sapiens-RNA-Seq
SRR1544502
SRR1544502_GSM1473812-Qui-2-Homo-sapiens-RNA-Seq
SRR1544481
SRR1544481_GSM1473764-Immortal-2-Homo-sapiens-RNA-Seq
SRR1544480
SRR1544480_GSM1473759-Immortal-1-Homo-sapiens-RNA-Seq
B
Output Files
NAME
..
SRR1544482.tar.gz
abundance.h5
abundance.tsv
generateOutputResults.html
run_info.json
