# Supplementary material for: Arkas: Rapid reproducible RNAseq analysis
Source: F1000Res. 2017 Jun 21;6:586. Originally published 2017 Apr 27. [Version 2] doi: 10.12688/f1000research.11355.2 (PMC5553089; doi:10.12688/f1000research.11355.2)
Supplement: Peer review history

# Peer review: Referee response for version 2

**DOI:** 10.5256/f1000research.12854.r23689

**Published:** 2017-08-07

**Authors:** Harold Pimentel

**Affiliations:** 1 Department of Genetics, Stanford University, Stanford, CA, USA

Hello Colombo *et al.*,

 Firstly: I am so very sorry for such a late review.

 Anyway, the new manuscript looks much better. Thanks for the revisions.

 I just have one nitpick: in Figure 2 you show the p-value distribution over the range (0, 0.05). Perhaps I missed something, but I'm not sure I completely understand the value of showing over this interval rather than the whole interval (0, 1). Does it have to do with the normalization adjusting p-values specifically in this range?

 Regardless -- nice work and congrats!

I have read this submission. I believe that I have an appropriate level of expertise to confirm that it is of an acceptable scientific standard.

# Peer review: Referee response for version 1

**DOI:** 10.5256/f1000research.12258.r22616

**Published:** 2017-05-24

**Authors:** Ted Abel, Marie Gaine

**Affiliations:** 1 Iowa Neuroscience Institute, University of Iowa, Iowa, USA

This paper introduces a RNA-Seq analysis pipeline, Arkas, which combines currently available tools typically used in RNA-Seq studies. The novelty of this pipeline is the encapsulation of tools needed to prepare the data, run quality control checks, analyze the data and perform secondary analyses. This is especially beneficial for investigators new to RNA-Seq analysis with little experience navigating through computational tools. The authors take care to outline the rationale behind creating an easy-to-use interface and how this will increase reproducibility and consistency across RNA-Seq studies.  They emphasize the importance of consistency with versions by showing differing results between two Kallisto versions.

 However, there are some minor limitations also found in this study:

 It would be beneficial to include quality control checks at the beginning of the pipeline to generate data regarding the inputted sequencing files.

 It would be interesting to see more processing time information to show the benefit of using this pipeline compared to similar methods.

 As is discussed, the inclusion of lncRNAs increases the amount of potentially interesting results from this pipeline. However, the authors have chosen to ignore microRNAs, an important regulator of cellular function. The inclusion of microRNAs as a default option in this pipeline would provide even more potentially interesting results.

 The normalization steps and Figure 2 should be discussed in more detail. Specifically, expand on the reasons for choosing these two methods and the differences between the methods and their outputs. In addition, a note about how a user should select a normalization type would help new users.

 Whilst the authors suggest that the integration of Docker will help produce reproducible research methods, the in-depth look into Docker is unnecessary, as no data has been provided to show its benefit above other options.

We have read this submission. We believe that we have an appropriate level of expertise to confirm that it is of an acceptable scientific standard.

# Response

**Published:** 2017-06-08

**Authors:** Giridharan Ramsingh

**Affiliations:** University of Southern California, USA

Thank you very much Dr. Abel for your insightful review. 

The revised manuscript removed the in-depth discussion of Docker because it was too broad.  The revised version included a discussion section that compares processing times between Google Genomics, and another BaseSpace application. 

Your comments helped address the analysis of microRNAs.  For example, Kallisto can process smaller FASTA sequences, however this invokes limitations to the construction of the Target DeBruijn Graph by increasing the path ambiguity of longer read sequences.  The revised manuscript now addressed this limitation, and suggested that users analyze microRNAs separately.  This analysis feature is not yet a default, but would be a great future addition.  We further address details in regard to normalization motivation and selection.

As suggested by the first reviewer Dr. Pimentel, we have significantly reduced the broad discussion section, and explicitly described the motivation for the development of *Arkas*.  We have additionally revised the 'Methods' section to provide a brief overview of the applications, and clearer descriptions of the interface style that included Supplementary Figures depicting both interfaces. 

*"This paper introduces a RNA-Seq analysis pipeline, Arkas, which combines currently available tools typically used in RNA-Seq studies. The novelty of this pipeline is the encapsulation of tools needed to prepare the data, run quality control checks, analyze the data and perform secondary analyses. This is especially beneficial for investigators new to RNA-Seq analysis with little experience navigating through computational tools. The authors take care to outline the rationale behind creating an easy-to-use interface and how this will increase reproducibility and consistency across RNA-Seq studies.  They emphasize the importance of consistency with versions by showing differing results between two Kallisto versions.*

*However, there are some minor limitations also found in this study:*

*It would be beneficial to include quality control checks at the beginning of the pipeline to generate data regarding the inputted sequencing files."*

Thank you for this suggestion.  Analyzing read quality will guide users into the important decision to filter low quality reads, however *Arkas *was not designed to address this.  In the revised manuscript, we have now mentioned another independent BaseSpace application *FastQC *which can assess read quality.  For users interested in manually uploading sequencing data to BaseSpace, each read must pass a quality filter.  This quality filter will automatically reject poor quality reads, and for this we designed *Arkas *with the assumption that sequenced reads input were of good quality. 

*"It would be interesting to see more processing time information to show the benefit of using this pipeline compared to similar methods."*

Thank you very much for addressing processing times. The revised manuscript significantly reduced the discussion section to comparisons of processing times.  Your remarks inspired the addition of processing times of *Arkas.  *We’ve included further information comparing the processing time to another BaseSpace application *RNAExpress. *Further, we added processing time information of a different Kallisto analysis pipeline implemented over Google Genomics Platform.  The discussion section now is far more concise with greater relevance toward the functionality of our developed software.   

*"As is discussed, the inclusion of lncRNAs increases the amount of potentially interesting results from this pipeline. However, the authors have chosen to ignore microRNAs, an important regulator of cellular function. The inclusion of microRNAs as a default option in this pipeline would provide even more potentially interesting results."*

Including microRNAs is a very great idea. *Arkas *can quantify microRNAs, but we decided not include microRNAs as default yet.  In the revised manuscript we address that the small sequence sizes are a potential limitation to quantification of cDNAs/ncRNAs because it may increase path ambiguities during the construction of the Target DeBruijn graphs.  Hence, we suggest that users analyze microRNAs separately and locally.  This would be a great additional feature for the next version of *Arkas.*

*"The normalization steps and Figure 2 should be discussed in more detail. Specifically, expand on the reasons for choosing these two methods and the differences between the methods and their outputs. In addition, a note about how a user should select a normalization type would help new users."*

Thank you for addressing this.  The revised manuscript has now explicitly stated how end-users may decide a selection of the normalization type.  We further provide a brief explanation to why unsupervised normalization was selected.  

*"Whilst the authors suggest that the integration of Docker will help produce reproducible research methods, the in-depth look into Docker is unnecessary, as no data has been provided to show its benefit above other options."*

 We agree that the discussion of Docker was too broad, and the revised discussion is focused on comparative performance from other cloud platforms.

# Peer review: Referee response for version 1

**DOI:** 10.5256/f1000research.12258.r22282

**Published:** 2017-05-18

**Authors:** Harold Pimentel

**Affiliations:** 1 Department of Genetics, Stanford University, Stanford, CA, USA

Note: I am a co-author of the kallisto tool, one of the tools that is used in this pipeline.

 Colombo et al. describe Arkas, a tool that takes raw RNA-Seq data and produces several different types of downstream analyses. Arkas leverages existing analysis tools (e.g. kallisto and limma) and platforms (Illumina BaseSpace) to create an easy to use, fast, and reproducible pipeline. A very useful (unique?) feature is that it documents software versions and enforces consistent software versions allowing users to see the potential differences with different software versions. This is made explicit in the "Results" section.

 Having all of these tools together greatly reduces the time to setup analyses and also reduces the complexity for RNA-Seq novices who might have no idea where to start. Arkas makes all of the typical figures one might make in a standard RNA-Seq analysis. It also provides gene-set analyses which are often excluded from other pipelines. In my experience, gluing together analyses from differential expression to gene-set analyses can often be an annoyance due to inconsistencies and annotations and versions of these annotations. Arkas nicely solves this problem.

 While I think the idea is very good and the tool seems comprehensive, I feel the manuscript needs a bit of work. Here are a few points:

 - There are a few areas where the scope seems too broad. In general, I feel that the manuscript can be shortened to be more clear as well as more precise. In particular, the Docker section in the discussion is too broad and the role of Arkas seems lost. I strongly recommend shortening this section and discussing the role of Docker in Arkas more clearly.

 - While the abstract and introduction provide a description of Arkas in RNA-Seq analysis, they do not provide a motivation. It is sort of hinted in several sections in the paper, but it is not explicit. The motivation of building another pipeline should be explicit.

 - How does this pipeline compare to other pipelines such as Galaxy, DNANexus, etc.? Should probably be noted in the introduction/discussion.

 - Perhaps I missed it, but the interface of Arkas does not appear to be described. There is a short subsection "Operation" that doesn't describe the type of interface. It appears to be available on Illumina BaseSpace, but does this make it a commandline tool or an online web form style tool? A short description of this interface and possibly supplementary figures (if it is a web form style) should be provided. This is unclear to folks who are not familiar with BaseSpace.

 - It should be greater emphasized how this tool can be used to reanalyze existing SRA data with relative ease. In my opinion this is a very strong argument as to why one might want a tool like this.

 Areas that can be shortened:

 - "Data variance between software versions" can be shortened as some of this is repeated in "Results."

 - "Complete transcriptomes enrich annotation information..." Specifics of annotations can probably be removed/condensed. It is probably sufficient to say that some are 3x times larger which can change results drastically.

 - "Docker as a cornerstone of reproducible research" The role of Docker in general can probably be shortened and how Arkas leverages it should be made more clear.

 More minor points:

 - A short sentence at the beginning of "Methods" should give an overview of the two-step process.

 - The Galaxy Project (https://usegalaxy.org/) should probably be cited even though the scope is a bit different.

 - Figure 1a: "Receiver Operator Characteristic plot" of what? This is stated in the main text, but should also the stated in the figure caption.

 - Swap Figure 1d and 1c.

 - It seems like BaseSpace sessions can easily be shared? If so, this is an additional strong point of using BaseSpace in Arkas.

 Overall, I'm very excited to see this comprehensive tool exist and be described in this paper.

I have read this submission. I believe that I have an appropriate level of expertise to confirm that it is of an acceptable scientific standard, however I have significant reservations, as outlined above.

# Response

**Published:** 2017-06-08

**Authors:** Giridharan Ramsingh

**Affiliations:** University of Southern California, USA

Thank you very much Dr. Pimentel for your thorough review.  We have significantly reduced the broad discussion section, and narrowed the manuscript to the most important features.  The 'Abstract' and 'Introduction' section was reduced to explicitly state the motivations for the design of *Arkas.  *In the revised manuscript, the 'Methods' section provides a brief overview of the applications, and the 'Operation' section describes the interface style and includes Supplementary Figures depicting both apps. 

The second reviewer Dr. Abel also suggested that the in-depth discussion of Docker was too broad.  The revised version includes a discussion section that is compares processing times between Google Genomics, and another BaseSpace application.  We also have now included brief points in regard to Galaxy.

Your helpful comments helped the manuscript become much more concise. In addition to your remarks, we have addressed important features regarding microRNAs on behalf of the second reviewer.  Kallisto can process smaller FASTA sequences, however we have now addressed that users can analyze microRNAs, but we suggest a separate analysis for this.

We thank you very much for your revisions and appreciate your thoughtful remarks.   We believe that addressing your remarks the manuscript is greatly elevated. Below are point-by-point responses to your questions.

*"Note: I am a co-author of the kallisto tool, one of the tools that is used in this pipeline.*

*Colombo et al. describe Arkas, a tool that takes raw RNA-Seq data and produces several different types of downstream analyses. Arkas leverages existing analysis tools (e.g. kallisto and limma) and platforms (Illumina BaseSpace) to create an easy to use, fast, and reproducible pipeline. A very useful (unique?) feature is that it documents software versions and enforces consistent software versions allowing users to see the potential differences with different software versions. This is made explicit in the "Results" section.*

*Having all of these tools together greatly reduces the time to setup analyses and also reduces the complexity for RNA-Seq novices who might have no idea where to start. Arkas makes all of the typical figures one might make in a standard RNA-Seq analysis. It also provides gene-set analyses which are often excluded from other pipelines. In my experience, gluing together analyses from differential expression to gene-set analyses can often be an annoyance due to inconsistencies and annotations and versions of these annotations. Arkas nicely solves this problem.*

*While I think the idea is very good and the tool seems comprehensive, I feel the manuscript needs a bit of work. Here are a few points:*

*- There are a few areas where the scope seems too broad. In general, I feel that the manuscript can be shortened to be more clear as well as more precise. In particular, the Docker section in the discussion is too broad and the role of Arkas seems lost. I strongly recommend shortening this section and discussing the role of Docker in Arkas more clearly.*"

Thank you very much for your input. In the revised manuscript, we have narrowed the Docker discussion section to the scope of BaseSpace platform, and have described *Arkas*' relationship to Docker as an applied infrastructure to this platform.  The previous version of the manuscript detailed the role of Docker in the broad concept of reproducible research.  We have omitted these details. The revised manuscript describes the interdependent relationship between Arkas and Docker in the context of BaseSpace.  For example, Arkas containerized Node.js and R to parse the BaseSpace JSON input information relating to BaseSpace’s input fields.  The new manuscript explained that Docker and Arkas are not independent entities, and pertain specifically to BaseSpace.

*"- While the abstract and introduction provide a description of Arkas in RNA-Seq analysis, they do not provide a motivation. It is sort of hinted in several sections in the paper, but it is not explicit. The motivation of building another pipeline should be explicit.*"

Thank you for this suggestion.  We have now explicitly provided the motivation for *Arkas*’ development by mentioning bottlenecks in RNA-sequencing such as sequencing importing and pre-processing steps, and how *Arkas *rectifies those bottlenecks.  In the revised version, we illustrate how *Arkas *was developed downstream from BaseSpace *SRA Import* to greatly reduce importing and conversion steps.  Also, we now explicitly stated the motivation for *Arkas-Quantification *such that Kallisto was implemented in parallel, which now scales quantification speed to the Amazon AWS EC2 cluster node availability rate.  In addition, the revised manuscript explicitly stated the motivation for *Arkas-Analysis, *which provides a comprehensive analysis.

*"- How does this pipeline compare to other pipelines such as Galaxy, DNANexus, etc.? Should probably be noted in the introduction/discussion.*"

Thank you for this suggestion.  In the revised discussion section, we now compare features of other cloud platforms, and other BaseSpace RNA-Seq applications.  The revised discussion now included processing times of a large scale RNA-seq analysis that implemented Kallisto using Google Genomics Platform.  In addition to Goolgle Genomics, the revised manuscript briefly compares features offered by Galaxy to BaseSpace.  Further we compare *Arkas *to other BaseSpace RNA-Seq applications.

*"- Perhaps I missed it, but the interface of Arkas does not appear to be described. There is a short subsection "Operation" that doesn't describe the type of interface. It appears to be available on Illumina BaseSpace, but does this make it a commandline tool or an online web form style tool? A short description of this interface and possibly supplementary figures (if it is a web form style) should be provided. This is unclear to folks who are not familiar with BaseSpace.*"

Thank you again for this suggestion.  We have included a description explicitly stating that Arkas is a web form style.  In addition, we included two Supplementary Figures to address the web input forms.  Supplementary Figure 1 shows the input form for both web style apps, and Supplementary Figure 2 shows the output folder directory of the *Arkas-Quantification*.

*"- It should be greater emphasized how this tool can be used to reanalyze existing SRA data with relative ease. In my opinion this is a very strong argument as to why one might want a tool like this.*"

Thank you for addressing reanalysis of SRA data.  In the updated manuscript, we now mention that *Arkas' *design was motivated by the BaseSpace application *SRA Import.  *The revised introduction now explicitly stated that *Arkas *is SRA compatible and we have provided citations for readers interested in utilizing this SRA application.  

*"Areas that can be shortened:*

*- "Data variance between software versions" can be shortened as some of this is repeated in "Results." '"*

We combined the “Data variance between software versions” and “Results” section into an appropriate concise section.

*- "Complete transcriptomes enrich annotation information..." Specifics of annotations can probably be removed/condensed. It is probably sufficient to say that some are 3x times larger which can change results drastically.*"

We reduced this discussion to brief specifics of database sizes.  While obvious, we believe that a brief overview provides motivation for the default transcriptomes chosen by *Arkas.  *In the revised manuscript, we provide a very concise explanation behind the selection of default transcriptomes.

*- "Docker as a cornerstone of reproducible research" The role of Docker in general can probably be shortened and how Arkas leverages it should be made more clear.*"

Thank you again for this comment.  We agree that this broad discussion went off topic and may distract future readers.  The manuscript is greatly improved with the removal of the discussion about democratization of research efforts, and biotechnology. We significantly revised the discussion to a comparison of differing cloud platforms and corresponding processing times of other cloud applications. 

*"More minor points:*

*- A short sentence at the beginning of "Methods" should give an overview of the two-step process.*"

We provided an overview of *Arkas *in the section described.

*"- The Galaxy Project (https://usegalaxy.org/) should probably be cited even though the scope is a bit different.*"

Galaxy is briefly mentioned in the discussion.  The revised manuscript reviewed and compared processing times of Google Genomics Platform and another RNAseq application within BaseSpace.

*"- Figure 1a: "Receiver Operator Characteristic plot" of what? This is stated in the main text, but should also the stated in the figure caption.*

*- Swap Figure 1d and 1c.*"

Thank you for pointing this out.  The revised Figure 1a now states that the Receiver Operator Characteristic plot is for ratios of detected and actual spiked ERCC sequences.  We have swapped Figure1d and Figure 1c.

*"- It seems like BaseSpace sessions can easily be shared? If so, this is an additional strong point of using BaseSpace in Arkas."*

 We now mention this brief point in the discussion.

*"Overall, I'm very excited to see this comprehensive tool exist and be described in this paper.*"

Thank you very much Dr. Pimentel.
